# Prevalence and trends of transfusion transmissible infections among blood donors in the State of Qatar, 2013–2017

**DOI:** 10.1186/s12879-020-05344-5

**Published:** 2020-08-20

**Authors:** Mohamed Aabdien, Nagah Selim, Sayed Himatt, Saloua Hmissi, Zeyd Merenkov, Noora AlKubaisi, Manar E. Abdel-Rahman, Abdelatif Abdelmola, Shadi Khelfa, Elmoubasher Farag, Hamad E. Al-Romaihi, Mohamed Al-Thani, Moutaz Derbala, Saad Al-Kaabi

**Affiliations:** 1 Community Medicine Training Program- Medical Education, Hamad Medical Corporation, P.O. Box 3050, Doha, State of Qatar; 2 Community Medicine Training Program- Primary Health Care Corporation, Doha, Qatar; 3 Faculty of Medicine- Cairo University, Cairo, Egypt; 4 Public Health Department- Ministry of Public Health, Doha, Qatar; 5 Blood Donation Unit- Hamad Medical Corporation, Doha, Qatar; 6 College of Health Sciences- Department of Public Health, Qatar University, Doha, Qatar; 7 Gastroenterology and Hepatology Department- Hamad Medical Corporation, Doha, Qatar

**Keywords:** Blood Donors, Transfusion, Hepatitis B virus, Hepatitis C virus, Human T-Lymphotropic virus, Syphilis, Malaria, Qatar

## Abstract

**Background:**

Millions of lives around the world are being saved annually through blood transfusion. However, blood transfusion is among the essential vehicles for transmitting infections. The overall prevalence of Transfusion Transmissible Infections among blood donors differs around the world, reflecting the variation in the prevalence of these infections. This study aims to assess the prevalence and trends of Transfusion Transmissible Infections among blood donors in Qatar.

**Methods:**

This is a cross-sectional study utilizing donation records of 5 years from January 2013 to December 2017. We included in the study results for all screening and confirmatory tests for Hepatitis B Virus, Hepatitis C Virus, Human T-lymphotropic Virus-I/II, Syphilis and Malaria.

**Results:**

Among the 190,509 donations received at the donation centre during the study period, about 91% of donations were received from males and 9% from females. The overall positivity rate for all tests was 1.87, 2.23, 1.78, 2.31, 2.67% for the years 2013 through 2017, with an increasing yearly trend by 6% each year. The overall positivity rates for Hepatitis C Virus, Human T-lymphotropic Virus-I/II, Hepatitis B Virus, Syphilis and Malaria (2013–2017) were 0.60, 0.18, 0.30, 0.43 and 0.20%, respectively.

**Conclusion:**

The overall positivity rate of all tests combined for the Transfusion Transmissible Infections demonstrated a gradually increasing trend from 2013 to 2017. However, the trend for each infection (Hepatitis C Virus, Hepatitis B Virus, Syphilis and Malaria) was fluctuating except for Human T-lymphotropic Virus-I/II, which was increasing. Supporting the development of effective prevention and control strategies requires further comprehensive investigations for better estimation of the burden of these infections.

## Background

Blood transfusion is a life-saving procedure that saves millions of lives every year around the world, it can be transfused as whole blood for one patient or may be manufactured into blood-derived products to be provided for more than one patient. However, it is known that blood transfusion can be associated with risks of transmitting certain infections [1, 2].

These infections are known as Transfusion Transmissible Infections (TTIs), which defined as any infection that can be transmitted from person to person through parenteral administration of blood or any blood products. Thus, different outcomes may follow unsafe transfusion, as it can cause an acute clinical sickness; it can persist in the receiver as a carrier or cause asymptomatic infection [3].

These infections include viral, bacterial, parasites and prions. The most prominent among these are, Human Immunodeficiency Virus (HIV), Hepatitis B Virus (HBV) and Hepatitis C Virus (HCV), due to their high prevalence rates. Other agents are Human T-cell Lymphotropic Virus (HTLV-I/II), Cytomegalovirus (CMV), Parvovirus B19, West Nile Virus (WNV) and Dengue Viruses, Trypanosomiasis, Malaria and Transmissible Spongiform Encephalopathy (TSE) [4].

The prevalence of TTIs among blood donations varies between high and low-income countries. It was reported that the prevalence of HIV, HBV, HCV and Syphilis in high-income countries are 0.003, 0.03, 0.02 and 0.05%, respectively. While in low-income countries, the prevalence of these infections is higher; 1.08, 3.70, 1.03 and 0.90%, respectively. These differences reflect the variation in the prevalence of these infections among the populations of these countries [5]. These variations are also observed between the World Health Organization (WHO) regions. As African and Pacific regions are the highest in HBV prevalence, Eastern Mediterranean region is the highest for HCV prevalence, Sub-Saharan Africa is the highest for malaria, and scattered foci in southwestern Japan, Colombia and intertropical Africa are endemic for HTLV-1 [6–8].

Many global efforts are provided to ensure the safety of the whole blood transfusion process. These efforts include providing recommendations and guidelines to establish a national blood screening and surveillance system for the entire transfusion chain, i.e. haemovigilance system. Moreover, efforts are also provided for the establishment of the Global Database on Blood Safety (GDBS), aiming to improve transfusion services globally [9, 10].

In Qatar, Expatriates comprised a high proportion of Qatar’s population, coming from all over the world, with diverse and unique epidemiological characteristics for each region. Enormous efforts aimed at protecting the health of the population and prevention the spread of infectious diseases from newcomers. Among these efforts is the law that mandates all newcomers wishing to work and live in Qatar to undergo a medical exam and infectious diseases screening to be able to receive a residence permit. Furthermore, specific job categories, including health care personnel and food industry workers, are required to undergo annual medical check-ups.

The Blood Donor Center at Hamad Medical Corporation (HMC) is responsible for the provision of safe blood supply through the efforts provided to ensure that donated blood is free from infections. However, few local epidemiological studies are assessing the prevalence and trends of transfusion-transmitted infections among blood donors, comprehensively. Hence, conducting this study to assess transfusion transmissible infections among blood donors can provide a better understanding of the epidemiology of these infections, which can support strategies development to evaluate safe blood supply measures, in addition to the preventive and control measures that are needed to manage the burden of these infections in the community. Therefore, the study aims to assess the prevalence and the trends of the transfusion transmissible infections among blood donors during the period 2013–2017, in Blood Donor Center at HMC in the State of Qatar.

## Methods

### Study setting

The Division of Transfusion Medicine includes the Blood Bank Section & Donor Unit. The Transfusion Division is the only provider of blood and blood components in the State of Qatar. Its main function is to assure the availability of safe blood whenever needed. ​ The centre follows international recommendations for the provision of safe, efficacious blood products and transfusion. In September 2017, HMC’s committee for blood transfusion announced a new program for monitoring blood transfusion process in the country. The Centralized Hemovigilance Program aims to improve the quality of the blood transfusion chain, with the main focus on safety.

The program was initially implemented at HMC and was introduced across all healthcare facilities around the country, that collect, use or store blood and its products. All donated unit samples undergo screening tests prior transfusion for the following infectious, through following methods: Chemiluminescent Microparticle Immunoassay (CMIA) by (ARCHITECT, Abbott laboratories) for detecting HIV Ag/Ab, HBsAg. In addition to antibodies for HTLV-I/II, Syphilis TP, HCV, HBsAg and anti-HBc. Confirmatory tests are performed through Line Immuno Assay (LIA) by (FUJIREBIO, Europe N.V.) as a confirmatory test for the presence of these antibodies. Moreover, (ARCHITECT, Abbott laboratories) Malaria Ag P.f/Pan and Enzyme-linked Immunosorbent Assay (ELISA) by (NOVA TEC IMMUNOIAGNOSTICA, GmpH) are used for determination of Plasmodium antigens and antibodies, respectively.

### Data collection

This is a cross-sectional study, conducted using data of all blood donations received in the period between January 2013 and December 2017 at Blood Donor Center in HMC. All data included in the study were retrieved from the records anonymously with no identifications of the donors. An extraction sheet was used, and data were provided only as frequencies of positive results for each screening test, in addition to basic demographics including age, gender and nationality of all donors. Tests included in this study were: HBsAg, HBcAb, HBV NAT, HCV Ab, INNO-LIA HCV, HCV NAT, HTLV-I/II Ab, INNO-LIA HTLV-I, Syphilis Ab and INNO-LIA Syphilis, in addition to Malaria Ab and Ag tests. Data for HIV were not included due to its sensitivity, complexity, need for careful handling and unique address with a specific and comprehensive approach.

### Data Analysis

Data were extracted from the records and entered to Microsoft excel. Frequencies and percentages were used for the description of blood donors by gender, age groups and nationality. Furthermore, the analysis was conducted to assess the positivity rate of the transfusion transmissible infections distribution by age groups, gender, and nationality (Qataris and Non-Qataris). Infections positivity rates were calculated for each year to identify trends throughout the 5 years. Categorical variables were compared using the χ^2^ test. Test for trend *p*-value was obtained using multivariable Poisson regression with robust variance modelling the rate of positive tests to year (as a continuous variable) adjusting for age and using the proportion of donors in the population as an offset. *P*-values less than 0.05 were considered statistically significant, and Stata/ SE version 15 was used to conduct the analysis.

## Results

**Fig. 1:**
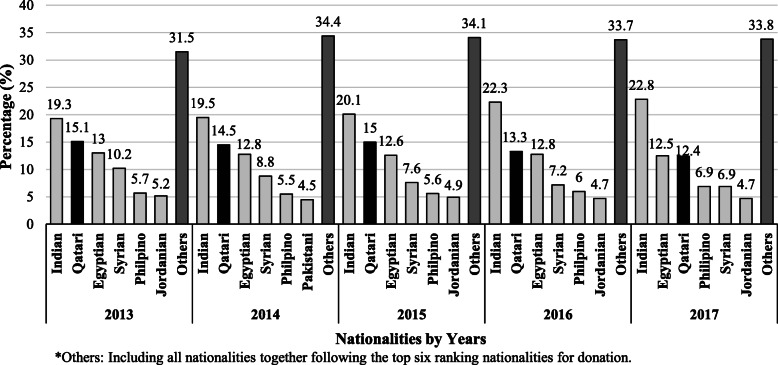
Percentage of blood donations by nationality in Blood Donors Center at HMC in the period 2013–2017. *Others: Including all nationalities together following the top six ranking nationalities for donation

A total of 190,509 donations were received at Blood Donor Centre in HMC in the period between January 2013 and December 2017. About 91% of the donations were received from males and 9% from females (Table 1). Moreover, donors of the age group 31–40 years were the highest to donate blood, with a percentage of 38.6% of the total donations during the study period (Table 1). Qataris were among the top six nationalities to donate blood in all years of the study (Fig. 1).

**Table 1:** Demographic characteristics of blood donors in Blood Donor Center at HMC in the period 2013 2017

Year	2013	2014	2015	2016	2017	Total
**Total Donations**	26,153	38,577	42,528	40,865	42,386	190,509
	**N (%)**	**N (%)**	**N (%)**	**N (%)**	**N (%)**	**N (%)**
**Gender**						
Male	24,140 (92.3)	34,961 (90.6)	38,531 (90.6)	37,028 (90.6)	38,577 (91.0)	173,237 (90.9)
Female	2013 (7.7)	3616 (9.4)	3997 (9.4)	3837 (9.4)	3809 (9.0)	17,272 (9.1)
**Age Group**						
≤ 20	770 (2.9)	1276 (3.3)	1194 (2.8)	1357 (3.3)	915 (2.2)	5512 (2.9)
21–30	9035 (34.5)	13,341 (34.6)	14,598 (34.3)	13,847 (33.9)	13,131 (31.0)	63,952 (33.6)
31–40	10,044 (38.4)	14,609 (37.9)	16,112 (37.9)	15,706 (38.4)	16,974 (40.0)	73,445 (38.6)
41–50	4831 (18.5)	6977 (18.1)	7920 (18.6)	7424 (18.2)	8381 (19.8)	35,533 (18.7)
51–60	1337 (5.1)	2142 (5.6)	2429 (5.7)	2264 (5.5)	2644 (6.2)	10,816 (5.7)
> 60	136 (0.5)	232 (0.6)	275 (0.6)	267 (0.7)	341 (0.8)	1251 (0.7)

**Fig. 2:**
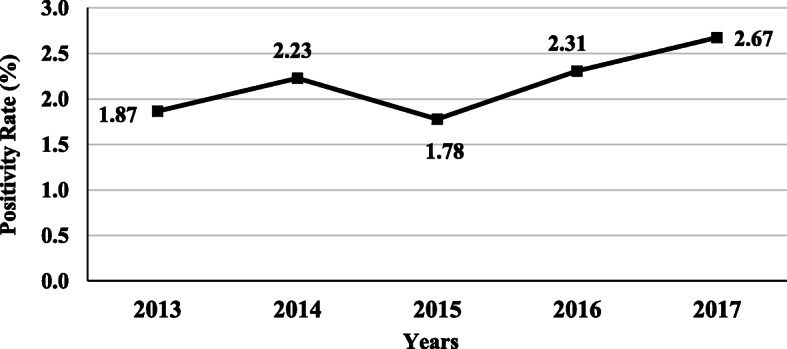
Temporal trend of total TTIs prevalence using combined serological and NAT screening (HBsAg, HBcAb, HBV NAT, HCV Ab, INNO-LIA HCV, HCV NAT, HTLV-I/II Ab, INNO-LIA HTLV-I, Syphilis Ab and INNO-LIA Syphilis, in addition to Malaria Ab and Ag tests) among blood donors in blood donors centre at HMC in the period 2013–2017

The positivity rates for all the tests combined (i.e. screening and confirmatory) were: 1.87, 2.23, 1.78, 2.31, 2.67% for the years 2013, 2014, 2015, 2016 and 2017, respectively (Fig. 2). Multivariable Poisson regression analysis showed that compared to the year 2013, the age-adjusted incidence rate ratio of positivity for years 2014, 2015, 2016 and 2017 were 1.24, 1.02, 1.24 and 1.32, respectively. The average age-adjusted rate of positives tests significantly increased by 6% each year (*P* <  0.001).

**Fig. 3:**
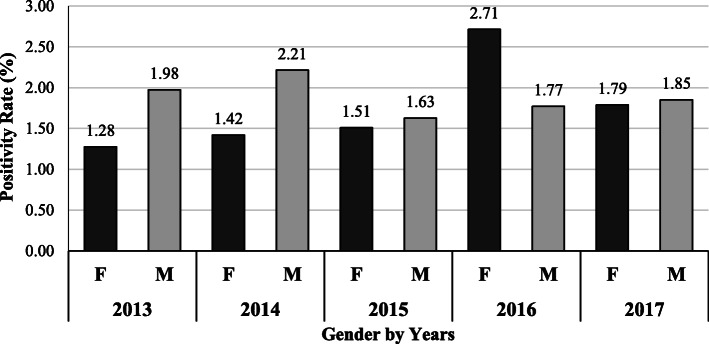
Gender distribution of positivity rate for combined serological and NAT screening (HBsAg, HBcAb, HBV NAT, HCV Ab, INNO-LIA HCV, HCV NAT, HTLV-I/II Ab, INNO-LIA HTLV-I, Syphilis Ab and INNO-LIA Syphilis, in addition to Malaria Ab and Ag tests) of blood donors in Blood Donors Center at HMC in the period 2013–2017

Furthermore, the positivity rates for tests combined were compared between male and female donors. Results showed that the rates were more among male donors in 2013 through 2015, which was significant only for 2013 and 2014 (*P* <  0.001). However, in 2016, the rates were significantly higher among females (*P* < 0.001), but the difference was again higher among male donors (*P =* 0.056) in 2017 (Fig. 3).

**Fig. 4:**
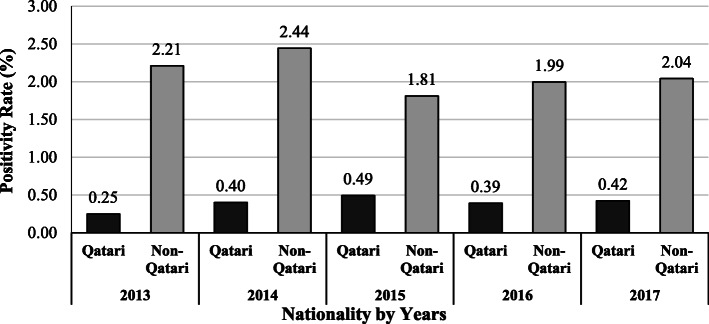
Positivity rate (%) for TTIs tests combined: (HBsAg, HBcAb, HBV NAT, HCV Ab, INNO-LIA HCV, HCV NAT, HTLV-I/II Ab, INNO-LIA HTLV-I, Syphilis Ab and INNO-LIA Syphilis, Malaria Ab and Ag tests) of blood donors (Qatari Vs Non-Qatari) in Blood Donors Center at HMC in the period 2013–2017

Assessing the positivity rates among age groups showed that the rates were higher among young donors, except for the years 2013 and 2014, in which the rates were higher in older age groups (*P* < 0.001), (Table 2). Moreover, when the rates were assessed according to nationality throughout the years, it was found that these rates were higher in Non-Qataris compared to Qataris throughout all the study period (*P* < 0.001), (Fig. 4).

**Table 2:** Age distribution of positivity rate (%) for TTIs combined tests of blood donors in Blood Donors Center at HMC, 2013–2017

Age Groups	≤ 20	21–30	31–40	41–50	51–60	> 60	P-value
**2013**	1.53	1.61	2.06	2.36	2.23	1.39	*P <* 0.001
**2014**	2.42	1.87	2.28	2.46	2.32	3.10	
**2015**	2.48	1.47	1.59	1.84	1.63	2.32	
**2016**	4.66	1.73	1.71	1.84	1.83	1.45	
**2017**	6.35	1.72	1.80	1.86	1.54	3.02	

Combined Tests: (HBsAg, HBcAb, HBV NAT, HCV Ab, INNO-LIA HCV, HCV NAT, HTLV-I/II Ab, INNO-LIA HTLV-I, Syphilis Ab and INNO-LIA Syphilis, in addition to Malaria Ab and Ag tests)

Furthermore, positivity rates of the screening tests were found to be fluctuating over time, for HBV (*P =* 0.256), Malaria (*P* = 0.523**),** Syphilis (*P* = 0.868) and HCV (*P* = 0.067). However, trends were increasing for HTLV I/II from 0.08 to 0.23 (χ^2^ = 17.563 and *P* < 0.001) as shown in (Table 3).

**Table 3:** Temporal trends positivity rate (%) for screening among blood donors in Blood Donors Center at HMC in the period 2013–2017

Year	2013	2014	2015	2016	2017	Overall	P-value
**HCV Ab**	0.58	0.58	0.56	0.60	0.70	0.60	0.067
**HTLV I/II Ab**	0.08	0.16	0.20	0.22	0.23	0.18	< 0.001
**Syphilis Ab**	0.48	0.49	0.35	0.41	0.45	0.43	0.868
**HBsAg**	0.34	0.28	0.31	0.26	0.29	0.30	0.256
**Malaria Ag**	0.14	0.24	0.42	0.10	0.10	0.20	0.523

Screening Tests: (HCV Ab, HBsAg, HTLV I/II Ab, Syphilis Ab and Malaria Ag)

## Discussion

Even though blood transfusion saves millions of lives every year, unsafe blood remains a threat to the spread of infections. Moreover, it’s also important to address the fact that safe blood is considered a universal right, and it should not cause any harm. Thus, it should be fully screened and ensured not be contaminated by any transmissible infection [11].

Blood donation centres are among valuable sources of data, as the prevalence of transfusion transmissible infections among blood donors differs throughout the world, and it can indirectly reflect the burden variations among these populations. This is because blood donors are usually representing the healthy members of the community so that it can have its inferences on the general population. Currently, the number of registered donors at Blood Donor Center in HMC had increased noticeably as a result of the continuous promotion, motivation, guidance, and education efforts about the importance of blood donation, which led to the overall rise in the general public awareness.

In this study, the predominance of blood donations was by males, which was consistent with several studies [12, 13]. Literature showed that women contribute less to blood donations than men due to many factors, including physiological factors such as menstruation, lactation and pregnancy [14]. However, some studies showed that this difference between gender is much less [15, 16].

The predominance of males’ contribution to blood donations was also observed in regards transfusion transmissible infections positivity rates, as this study showed a consistent predominance throughout most of the years, in which rates were higher in male donors. These were similar to the findings from studies in the region [17–20].

In general, the younger population seems to contribute more to blood donation, as it was shown in several studies, in which most donors were less than 30 years in age [12, 21, 22]. However, in this study, most of the blood donors found to be aged 31–40 years old. Additionally, positivity rates of infections were found to be more in the younger population, which was also found in other studies conducted in countries from different regions [21–23].

In literature, it has been discussed that young males are more to be involved in risky behaviours than females and older age groups. Moreover, results from several studies showed that some donors are involved in risky behaviour activities, yet they contribute to blood donation. The reason is that they use it to check if they were infected with any of the infections that can be related to their risky behaviours [24–26].

In this study, the positivity rates of the combined tests, including serological and NAT tests, were assessed throughout the years 2013 to 2017. By assessing these rates during this period, we were able to identify the trend for these infections, which found to be an increasing trend, after adjusting to age and the total population. Despite this increasing trend, yet, the rates were less than what was found in countries from different regions, such as Saudi Arabia, Egypt, India and Nigeria [27–30].

Moreover, the positivity rates were also assessed for selected tests throughout the study period. The results showed a fluctuating trend for HBsAg, between 0.34 in 2013 and 0.29 in 2017. These rates are less than what has been found in studies from countries in the region including Saudi Arabia, Kuwait, Jordan and Egypt [31], which can be attributed to the effective prevention and control strategies including vaccination programs [32].

The rates for HCV Ab showed a changing trend between 0.58 in 2013 and 0.70 in 2017. However, these rates are less than the reported from Egypt and Saudi Arabia [33], which can be attributed to the high prevalence of the disease among the general population and the migration from these countries to other countries with lower prevalence [34]. This was consistent with findings from our study, as the rates were higher among the Non-Qataris throughout the years. However, the rates were higher than what was reported from other countries in the same region (i.e. middle east region), like Iran and Turkey [33].

Furthermore, screening for HTLV is also among the recommended screening for transfusion, and this includes two viruses: I and II that differs in their geographical distribution and clinical disease association. HTLV is endemic in some parts of the world, and in some regions, the prevalence of the virus is very low, as the situation here in Qatar.

The cost-effectiveness of the universal screening strategies for HTLV has been argued. This was especially for the screening implementation by high-income countries with a low prevalence of the infection [35, 36]. Qatar is among these countries. However, it is a destination for working force from all around the world, including areas endemic with the virus. Therefore, HTLV is among the screening requirements for blood safety in the country. The positivity rate for HTLV I/II Ab in 2017 found to be 0.23, which represents an increasing trend from 0.08 in 2103. Further analysis for the findings showed that the positivity rates were more in Non-Qataris than Qataris for the screening test, but zero positivity among Qataris for the confirmatory test (i.e. INNO-LIA), which is consistent with epidemiological distribution of the disease, considering Qatar as low prevalence area for HTLV [7].

Donors can threaten blood safety with risky behaviour as they may potentially acquire syphilis, among other infections. In this study, the positivity rates for Syphilis Ab among donors throughout the years showed a fluctuating trend that reported to be 0.48 in 2013 and to be 0.45 in 2017, and these rates were higher in Non-Qatari throughout the years. Yet, these rates were less than what was found in studies in neighbouring countries, such as Saudi Arabia [12, 37]. Moreover, it’s important to consider the fact that a specific assay has been used to screen for syphilis (i.e. Treponema pallidum Ab), which leads to identifying whoever has been infected with syphilis, whether it is a recent or past infection, and whether it was treated or not, which may result in overestimation of the disease burden [35].

Another serious infectious agent that remains to risks blood safety is transfusion-associated malaria. The country is free from local transmission of malaria, and all the reported cases were imported from abroad, through migrants and travellers from endemic countries. Findings from our study are consistent with the situation among the general population, as the rates of positivity for Malaria Ag were found to be higher among Non-Qataris throughout the years. Moreover, assessing these rates throughout the year revealed a fluctuating trend. Additionally, the overall positivity rate to the regional countries showed similar rate to Saudi Arabia, but higher than the United Emirates and less than Pakistan [38, 39].

Finally, it is essential to highlight the fact that the variance in the prevalence of the transfusion transmissible infections among blood donors from different countries can be a reflection of an actual variance of the prevalence in these countries, in addition to the different methods used to test for these infections which can result in different estimations [40, 41].

In summary, the overall positivity rate of transfusion transmissible infections among blood donors was assessed through combined serological in addition to NAT tests, and results revealed a gradually increasing trend throughout the years, with 6% increase for each year. However, this study also revealed fluctuating trends for HBV, HCV, Syphilis and Malaria. In addition to the increasing trend for HTLV. Further analysis revealed that rates were higher among Non-Qataris throughout the years.

Despite the important study findings, some limitations are worth to be mentioned. Among which is the fact that this is a retrospective study conducted by utilizing records in the blood donor centre. The study was limited to the available data in these records only, which included tabulated results of the screening tests by basic demographics (i.e. age, gender, nationality). Population data was not available for all basic demographics’ categories, thus limiting some of the analysis that accounts for populations changes. Moreover, the study would have benefited from a longer period exceeding the current period from 2013 to 2017. Another limitation is not including data about HIV, due to the sensitivity and the complexity of these data. Finally, it was not possible to assess the outcome of donors with positive results for any of the infections, as the donation records are not connected with the medical records.

## Conclusion

In spite of the previously discussed limitations, this study still provides important findings that contribute to a better understanding of TTIs epidemiology. Up to our knowledge, this is the first study to assess the prevalence and trends of TTIs, including HBV, HCV, HTLV, Syphilis and Malaria among blood donors in Qatar. Further investigations are needed to assess the distribution and determinants of these infections in the community to support the development of effective prevention and control strategies and to protect the community from potential risks.

## Data Availability

The data that support the findings of this study are available from Blood Donation Center at HMC and were used under license for the current study, and so are not publicly available. Data are however available from the authors upon reasonable request and with permission of Blood Donation Center.

## Abbreviations

Anti-HBc: Antibody to Hepatitis B core antigen
CMIA: Chemiluminescent Microparticle Immunoassay
CMV: Cytomegalovirus
ELISA: Enzyme-linked Immunosorbent Assay
GDBS: Global Database on Blood Safety
HBsAg: Hepatitis B surface Antigen
HBV: Hepatitis B Virus
HCV: Hepatitis C Virus
HIV: Human Immunodeficiency Virus
HMC: Hamad Medical Corporation
HTLV-I/II: Human T-cell Lymphotropic Virus
LIA: Line Immuno Assay
TSE: Transmissible Spongiform Encephalopathy
TTIs: Transfusion Transmissible Infections
WHO: World Health Organization
WNV: West Nile Virus
